# The low‐field NMR studies the change in cellular water in tilapia fillet tissue during different drying conditions

**DOI:** 10.1002/fsn3.2221

**Published:** 2021-03-11

**Authors:** Jing Luo, Min Li, Ying Zhang, Man Zheng, Chang Ming Ling

**Affiliations:** ^1^ College of Food Science and Technology Guangdong Ocean University Zhanjiang China; ^2^ College of Mechanical and Power Engineering Guangdong Ocean University Zhanjiang China; ^3^ Guangdong Provincial Key Laboratory of Aquatic Product Processing and Safety Guangdong Ocean University Zhanjiang China

**Keywords:** bound water, cell membrane rupture, entrapped water, free water, low‐field nuclear magnetic resonance (LF‐NMR) technology, tilapia fillet

## Abstract

The muscle is a highly organized tissue, where there are three different moistures including free water, entrapped water, and bound water. These moistures were distributed in intercellular spaces, intracellular spaces, and other solute environments, respectively. Understanding the moisture migration in different environments is crucial to enhance energy efficiency and improve the quality of processed food. Therefore, the tilapia fillets were used to experiment, and the low‐field nuclear magnetic resonance technique is used to measure the change in different moistures during the drying process. The study found that free water is the highest when cell membranes started to rupture. In addition, it also observed that the cell membrane ruptures at different stages of drying. The result of this study provides critical information that could be used to guide the study of the dynamic mechanisms underlying drying and the development of drying technology for tilapia fillets and similar aquatic products.

## Graphical abstract

With the progress of drying, the surface and core temperature of the tilapia fillets continues to rise which leads to the decline of free water content, in addition, it was found that the free water is the highest when cell membranes started to rupture by the low‐field nuclear magnetic resonance technique. As the temperature increasing, the free water content appears a peak because the exposed intracellular water is able to readjust rapidly to become free water. The cell membrane ruptures at different stages of drying.

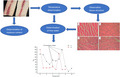

## 1 INTRODUCTION

Tilapia has the advantages of hypoxia tolerance, disease resistance capacity, rapid growth, and reproduction, and its nutritional value is high (Wang et al., 2019). In recent years, China became the top producers in the world to cultivate tilapia (FAO, 2017). In addition, in global fish farming, tilapia is the most popular farmed fish (Duan et al., 2011). But tilapia has high water content, and tilapia is apt to be highly spoilage when it is affected by enzymes and microorganisms (Kituu et al., 2010; Li et al., 2017), which can potentially result in substantial economic losses. In order to increase tilapia shelf life and maintain the quality of the tilapia products, it is of great necessary to dry for tilapia. At present, the methods of drying tilapia mainly include hot air‐drying (HAD), microwave drying (MD), hot air–microwave combined drying (HAMCD), heat pump drying (HPD), vacuum microwave drying (VMD), and vacuum freeze‐drying (VFD). Among these methods, the vacuum freeze‐drying (VFD) is the best method of water removal for all kinds of foods, but its operation cost is high (Duan et al., 2010). Therefore, it is limited to use. Vacuum freeze‐drying (VFD) is only used when it provides a reasonable added value to the products or the costly materials are dried (Ratti, 2001). In order to improve the quality of the products and reduce the drying time, it is necessary to pretreat the samples and combine two drying techniques to dry tilapia fillets. The previous research found that the quality of tilapia fillets by ultrasound‐assisted polydextrose osmotic vacuum freezing–heat pump combined drying (UAPOVFHPCD) is close to the quality of that by vacuum freeze‐drying; moreover, compared with vacuum freeze‐drying, it took less time. In addition, compared with heat pump drying (HPD), the quality of tilapia fillets by ultrasound‐assisted polydextrose osmotic heat pump drying (UAPOHPD) is better (Luo et al., 2020). In order to further improve product quality and save energy, therefore, it is very necessary to study the moisture water migration during UAPOVFHPCD and UAPOHPD. However, the drying process of materials is a very complex process, due to material complexity in internal structure and simultaneous heat and mass transfer when materials are dried (Esfahani et al., 2014; Khan et al., 2016). There are three different waters in the muscle; in addition, the proportion of moisture content in intercellular environments, intracellular environments, and other solute environments is different (Khan et al., 2017). The moisture in capillaries or intercellular spaces is referred as free water (Khan, Wellard, et al., 2017); the moisture in intracellular spaces is referred as intracellular water, sometimes also referred as entrapped water; and the moisture in other solute environments is referred as bound water (Khan, Wellard, et al., 2016). The moisture in aquatic products mainly exists in cells. Understanding the transfer of three waters (bound water, entrapped water, and free water) in aquatic product materials is essential during drying that helps to improve the quality of dried products.

At present, there are several techniques that are available for the determination of moisture content including differential scanning calorimetry (DSC), dynamic thermal mechanical analysis (DMTA), near‐infrared spectroscopy (NIR), and nuclear magnetic resonance (NMR) methods. However, the operation process in DSC, DMTA, and NIR is more complicated and cannot accurately reflect the spatial distribution and binding status of moisture (Chen et al., 2015). Nuclear magnetic resonance (NMR) can be applied to all kinds of raw materials without altering the sample or producing hazardous wastes. Magnetic resonance imaging (MRI) allows visual observations of the spatial and molecular distribution of water in food to permit monitoring of internal compositional and structural modification of foods during drying (Marcone et al., 2013). In NMR spectroscopy, the water content and water distribution in foods are known by the signal of water in food samples (Otero & Préstamo, 2009; Pearce et al., 2011). In addition, the NMR relaxation time can analyze the food ingredient, at the same time insight into food structure. Therefore, this method was successfully used to investigate the water distribution in different aquatic products, such as tilapia (Wang et al., 2020), sea cucumber (Tan et al., 2018), Sardinella brasiliensis (Carla et al., 2016), and hake fish (Sanchez‐Alonso et al., 2014), but these researches have not investigated the cellular water distribution in aquatic product tissue. Currently, it is very rare to investigate the migration mechanisms of bound water and free water during drying of aquatic products by low‐field NMR technology, and the study of changes in cell moisture by low‐field nuclear magnetic resonance technology mainly focus on agricultural products, for instance, by the NMR technology to investigate the changes in the extracellular and extracellular moisture in apples and potatoes during the drying process, and the investigation found that when the sample temperature exceeds 50℃, the cell membrane ruptured, and the intracellular moisture flows out of the cell (Khan et al., 2018; Khan & Karim, 2017). NMR T_2_ relaxometry is used to investigate the moisture content of the intracellular and extracellular in different fruits and vegetables (Khan, Wellard, et al., 2016). NMR is used to investigate the moisture content of the intracellular and extracellular in potatoes at lower temperatures and high temperature, and the investigation found that the cell membranes are intact when potatoes were dried at lower temperatures, but the cell membranes rupture at temperatures above 52℃ (Amit et al., 2011). In addition, the nuclear magnetic resonance (NMR) T_2_ relaxometry also had been used to investigate cellular water transport mechanism of animal lung (Sedin et al., 2000), brain (Sulyok et al., 2001), and liver (Moser et al., 1996).

Although many researchers have studied the free and bound water migration mechanism in plant‐based food material during drying, it is very rare to study changes in cell moisture in aquatic products by low‐field NMR technology, especially, which never appears in tilapia processing. Most researchers only investigate the relationship between different water T_2_ relaxation times and the percentage of moisture loss in aquatic products during drying. Therefore, the major aim of this study was to investigate the different water migration mechanisms in tilapia materials during different drying, which can improve energy efficiency and quality of processed food.

## 2 MATERIALS AND METHODS

### 2.1 Materials and reagents

Tilapias were purchased from a local Hu Guang Market in Ma Zhang District, Zhanjiang, China. Food‐grade polydextrose was purchased from Hebei Bai Wei Biotechnology Limited Company, China. NaCl was purchased from Xi Long Science Limited Company, China.

### 2.2 Test equipment

**FIGURE 1:**
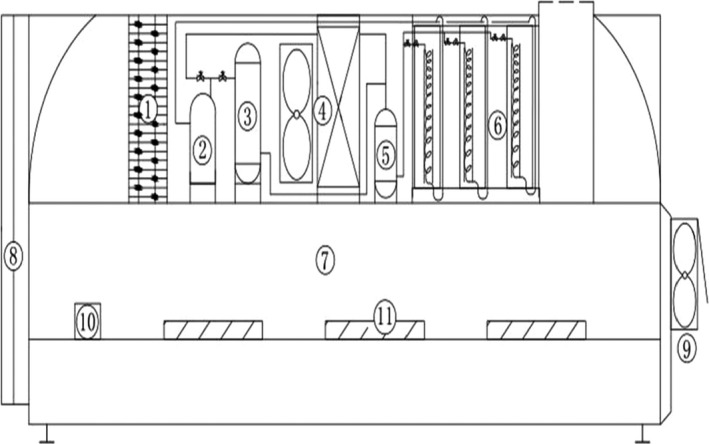
Heat pump drying device (①—auxiliary heater; ②—compressor; ③—water‐cooled condenser; ④—wind condenser; ⑤—liquid storage tank; ⑥—evaporator; ⑦—drying box; ⑧—movable door; ⑨—exhaust fan; ⑩—temperature, humidity, and wind speed test probes; and ⑪—test materials)

A schematic diagram of the heat pump drying device is shown in Figure 1. The heat pump device consisted of an auxiliary heater, a compressor, a water‐cooled condenser, a cold air condenser, a liquid storage tank, an evaporator, a drying oven, a movable door, an exhaust fan, a temperature probe, a humidity probe, a wind speed probe, and a test material group. The temperature of the auxiliary heater control equipment was in the range of −20–80℃ with an accuracy of ± 0.5℃. The humidity of the evaporator control equipment was in the range of 20%–80% with an accuracy of ± 5%. The wind speed was controlled by the variable frequency control of the exhaust fan motor, which was between 0.5 and 3 m/s with an accuracy of ± 0.1 m/s. The LGJ‐10E freeze dryer was purchased from Beijing Sihuan Scientific Instrument Factory Limited Company, China. The LGJ‐10E freeze dryer was used to dry the tilapia fillets [standard cold trap temperature (no load): ≤ −60℃ (ambient temperature ≤ 30℃), limit cold trap temperature (no load): ≤ −65℃ (ambient temperature ≤ 25℃), maximum water catching capacity: 3 kg, total power: 1,250 W]. The tilapia fillets were pretreated before being dried by the heat pump drying system and vacuum freeze‐drying system. The KQ‐500DE CNC ultrasonic cleaner was purchased from Kunshan Ultrasonic Instrument Limited Company, China. The total power of the KQ‐500DE CNC ultrasound cleaner was 500 W and was used to establish the ultrasound pretreatment conditions.

### 2.3 Sample processing

Fresh tilapia raw materials weighed approximately 0.9 kg. The meat on both sides of the back of the tilapia was first cut into pieces of size 100 mm × 50 mm × 5 mm with each weighing approximately 30 g (Li et al., 2012). The tilapia fillets were then permeated by ultrasound‐assisted polydextrose, followed by drying with the heat pump drying system and vacuum freeze‐drying system.

### 2.4 UAPOHPD

The optimal pretreatment conditions of the UAPOHP‐dried tilapia fillets were determined by preliminary experiments, using the following parameters: ultrasound power of 400 W, ultrasound time of 65 min, and polydextrose concentration of 60 g/L. Following pretreatment of tilapia fillets under these conditions, fillets were placed in a heat pump drying device under a temperature of 45℃ and wind speed of 2.5 m/s for heat pump drying until the dried basis moisture content fell to 0.3 ± 0.02 g/g, and the drying was complete (Li et al., 2012).

### 2.5 UAPOVFHPCD

The optimal pretreatment conditions of the UAPOVFHPC‐dried tilapia fillets were determined by preliminary experiments, using the following parameters: ultrasound power of 450 W, ultrasound time of 65 min, and polydextrose concentration of 80 g/L. Following pretreatment of tilapia fillets under these conditions, they were frozen for 2 hr under a temperature of −60℃ (until the central temperature of the fillets reached −20℃). The tilapia fillets were then dried for 5 hr by the vacuum freeze‐drying system under a 36℃ clapboard temperature and a 10‐Pa vacuum. Tilapia fillets were then placed in a heat pump drying device under an air temperature of 45℃ and a wind speed of 2.5 m/s until the dried basis moisture content fell to 0.3 ± 0.02 g/g, and the drying was complete.

### 2.6 Determination of initial moisture content

A certain amount of fresh fish meat was dried in the vacuum drying oven at 105 ℃ until it had completely dried. The initial moisture content was calculated according to Equation (1):

(1) $$W_{0}=\left(m_{0}‐m_{1}\right)/m_{0}\times 100\%$$

where W_0_ is the initial moisture content of the fish fillet (%); m_0_ is the quality of the fish fillet (g); and m_1_ is the absolute quality of the fish fillet (g).

### 2.7 Determination of the dried basis moisture content

During the drying process, the weight of the sample was weighed every 1 hr until the dried basis moisture content fell to 0.3 ± 0.02 g/g, and the drying was complete. The dried basis moisture content was calculated using Equation (2):

(2) $$W_{t}=\left[m_{t}‐m_{0}\times \left(1‐w_{0}\right)\right]/m_{0}\times \left(1‐w_{0}\right)$$

where W_t_ is the dried basis moisture content of the fish fillet at time t (g/g); m_t_ is the mass of the fish fillet at time t (g); m_0_ is the mass of the fresh fillet (g); and W_0_ is the initial moisture content of the fresh fillet (%).

### 2.8 Plotting drying curve

The dried basis moisture content of the fish fillets was measured every 1 hr, and the relationship of the dried basis moisture content with drying time using different drying methods was plotted.

### 2.9 LF‐NMR detection

#### 2.9.1 The acquisition and inversion of T_2_

The moisture distribution of the fish fillets was measured every 1 hr with the NMR analysis software. Tilapia fillets were placed in the center of the coil for nuclear magnetic testing, and the center frequency of the sample was obtained by an FID sequence. The main parameters of the T_2_ test included SF (MHz) = 21, RFD (ms) = 0.020, O_1_ (Hz) = 120,912.26, RG1 (db) = 20.0, P1 (us) = 13.00, DRG1 = 1, TD = 40,020, DR = 1, PRG = 0, TW (ms) = 3,500.00, NS = 16, P2 (us) = 26.00, TE (ms) = 0.250, and NECH = 8,000, peak offset(ms) = 0.0000, and phase count = 1. After the information is collected, the inversion dialog box reconstruction algorithm SIRT is used to perform 1 million iteration calculations to obtain the transverse relaxation time T_2_ of the samples. The experiments were done with three parallel samples. In addition, when the moisture distribution of the fish fillets was measured with the LF‐NMR equipment, the power supplies of all laboratory equipments were turned off to improve the signal‐to‐noise ratio. Increasing the numbers of NS can also reduce the effect of noise on the experimental results; however, if the NS is too large, the signal will overflow and the experimental results are affected. The signal intensity of LF‐NMR is related to the moisture content of samples, which is a proportional relationship. By comprehensively considering the signal of the highest and the lowest, the signal is the best at the NS = 16.

#### 2.9.2 The NMR imaging experiments and parameter setting

Tilapia fillets were placed in the center of the coil for NMR imaging testing, and the main parameters of the magnetic resonance imaging (MRI) were average = 3, slice count = 5, slice thickness (mm) = 2.99, and slice gap (mm) = 0.59, FOV = 150, waiting time (TR) = 500 ms, echo time = 20 ms, phase encoding steps = 192, and read size = 256.

#### 2.9.3 Data processing

The experimental data were drawn by Origin 8.0 software, and MRI images were drawn by the Niumag NMR image processing software v3.0.

## 3 RESULTS AND DISCUSSION

### 3.1 The effect of different drying methods on the drying curves of tilapia fillets

**FIGURE 2:**
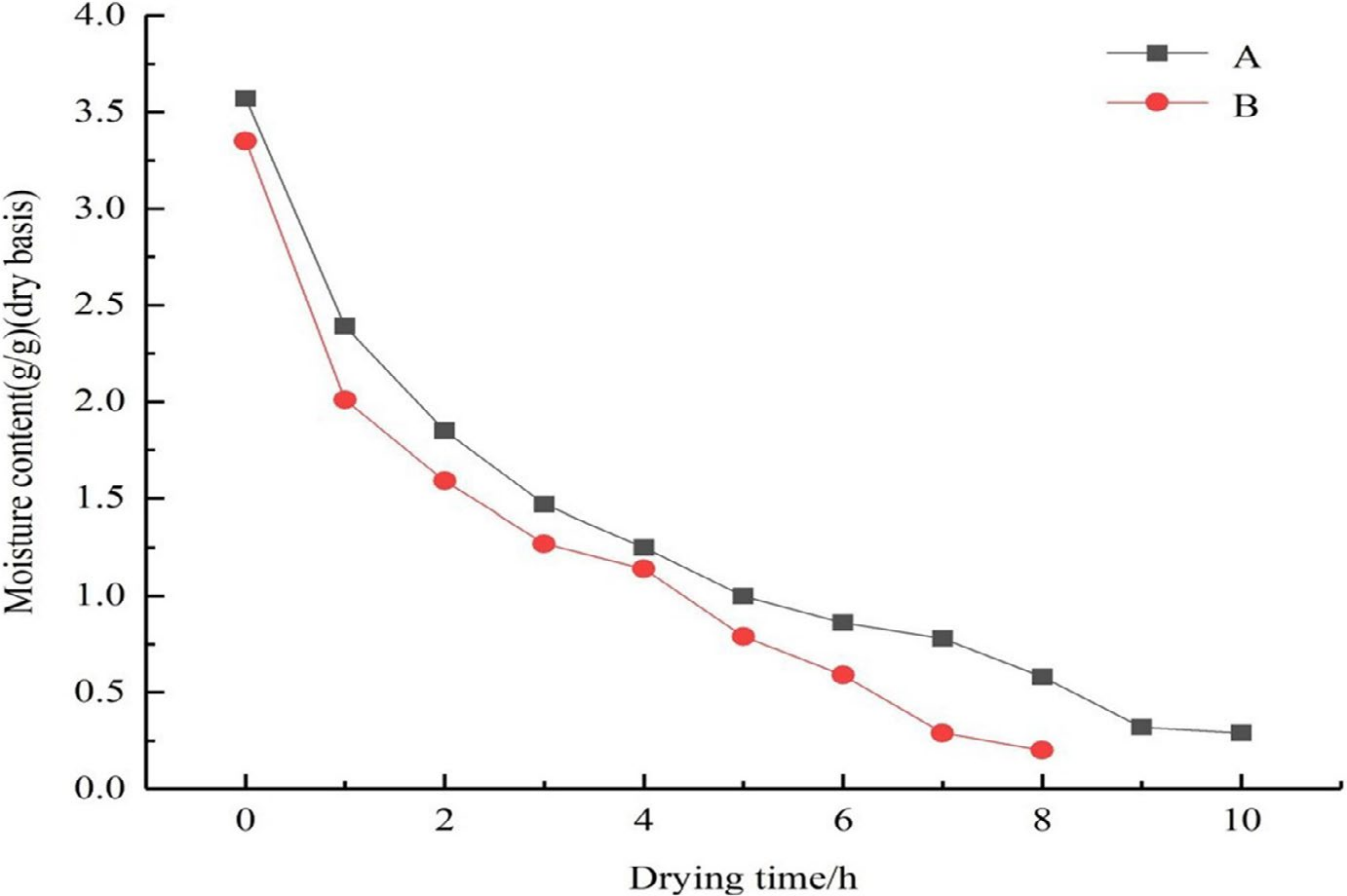
Drying curve of tilapia fillets under different drying conditions ((a) UAPOHPD; (b) UAPOVFHPCD)

The drying curves of tilapia fillets under different drying methods are shown in Figure 2. From Figure 2a, it can be observed that the initial and final moisture contents of tilapia fillets by UAPOHPD are 3.57 g/g and 0.29 g/g, respectively. From Figure 2b, it can be observed that the initial and final moisture contents of tilapia fillets by UAPOVFHPCD are 3.35 g/g and 0.2 g/g, respectively. It is depicted from Figure 2 that the dried basis moisture content of materials gradually decreased as drying time increases, and the drying rate of tilapia fillets by UAPOVFHPCD was more rapid relative to that achieved by UAPOHPD. Because tilapia fillets were dried using an UAPOVFHPCD technology, the internal structure of the materials formed gaps in the early stages of vacuum freeze‐drying, which was conducive to the diffusion of moisture during later stages of heat pump drying (Liu et al., 2012). However, after ultrasound‐assisted polydextrose osmotic pretreatment, the tilapia fillets were dried by a single bout of heat pump drying. As the drying time increased, the tilapia fillets hardened on the surface, which hindered the transport of internal moisture, reduced the rate of moisture diffusion, and increased the time required for the drying process to be completed (Rahman et al., 2016; Wang et al., 2011; Wei et al., 2020).

### 3.2 LF‐NMR spectra of tilapia fillets under different drying conditions

**FIGURE 3:**
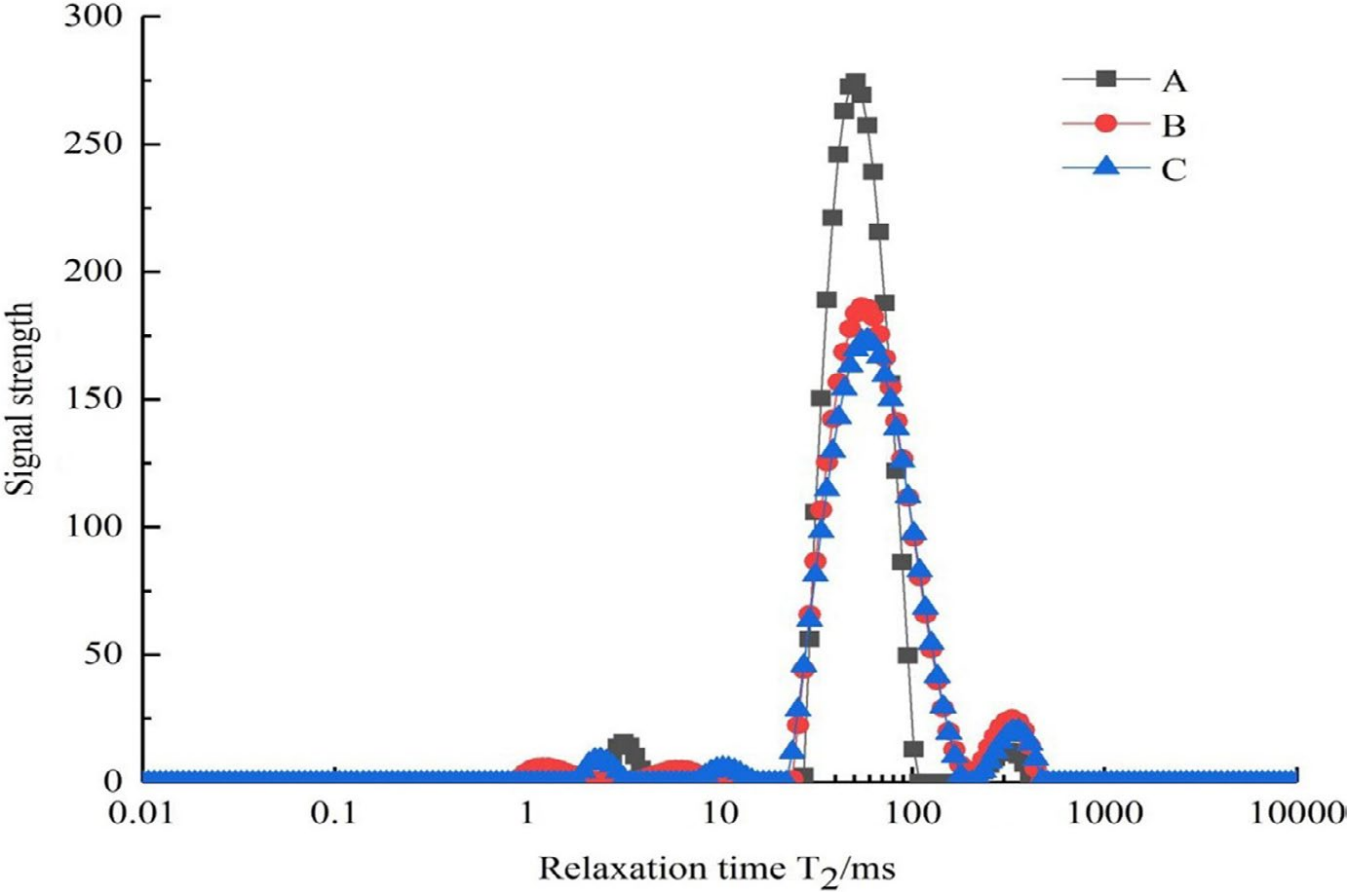
The transverse relaxation time T_2_ of different samples ((a) fresh fish fillets; (b) tilapia fillets were pretreated under ultrasonic power of 450 W, ultrasonic time of 65 min, and polydextrose concentration of 80 g/L; and (c) tilapia fillets were pretreated under ultrasonic power of 400 W, ultrasonic time of 65 min, and polydextrose concentration of 60 g/L)

The T_2_ inversion diagrams of fresh tilapia fillets are shown in Figure 3a, and its initial moisture percentage is 79%. From Figure 3, it can be observed that four peaks in the inversion NMR were visible, with each corresponding to the three water states. The ranges for the T_2_ ranges were T_21_ (0.01–10 ms), T_22_ (10–200 ms), and T_23_ (>200 ms). T_21_ refers to moisture that was tightly bound to the protein. T_22_ refers to the entrapped water in myogenic cells. T_23_ refers to the free water between myofibrils (Bertram & Andersen, 2007; Bertram et al., 2009; Fennema, 1985; Huff‐Lonergan & Lonergan, 2005; Offer & Knight, 1988). In addition, the corresponding peak area represents the relative content of water in each phase. In different environments, the proportion of three waters is different. The water about 85% is located in intracellular spaces (Huff‐Lonergan & Lonergan, 2005) and 15% is located in intercellular spaces (Hamm, 1975; Lawrie, 1998). The water in intracellular is easily removed, but the water in intercellular changes most obviously when the tissue structures are changed. Figure 3 shows that after ultrasound‐assisted osmotic pretreatment of polydextrose, the entrapped water in tilapia fillets noticeably decreased. The repeated compressing and stretching during the ultrasound treatment process resulted in a sponge effect and increased the intercellular space, which facilitated the discharge of internal water (Fernandes et al., 2009; Malgorzata & Malgorzata, 2016).

**FIGURE 4:**
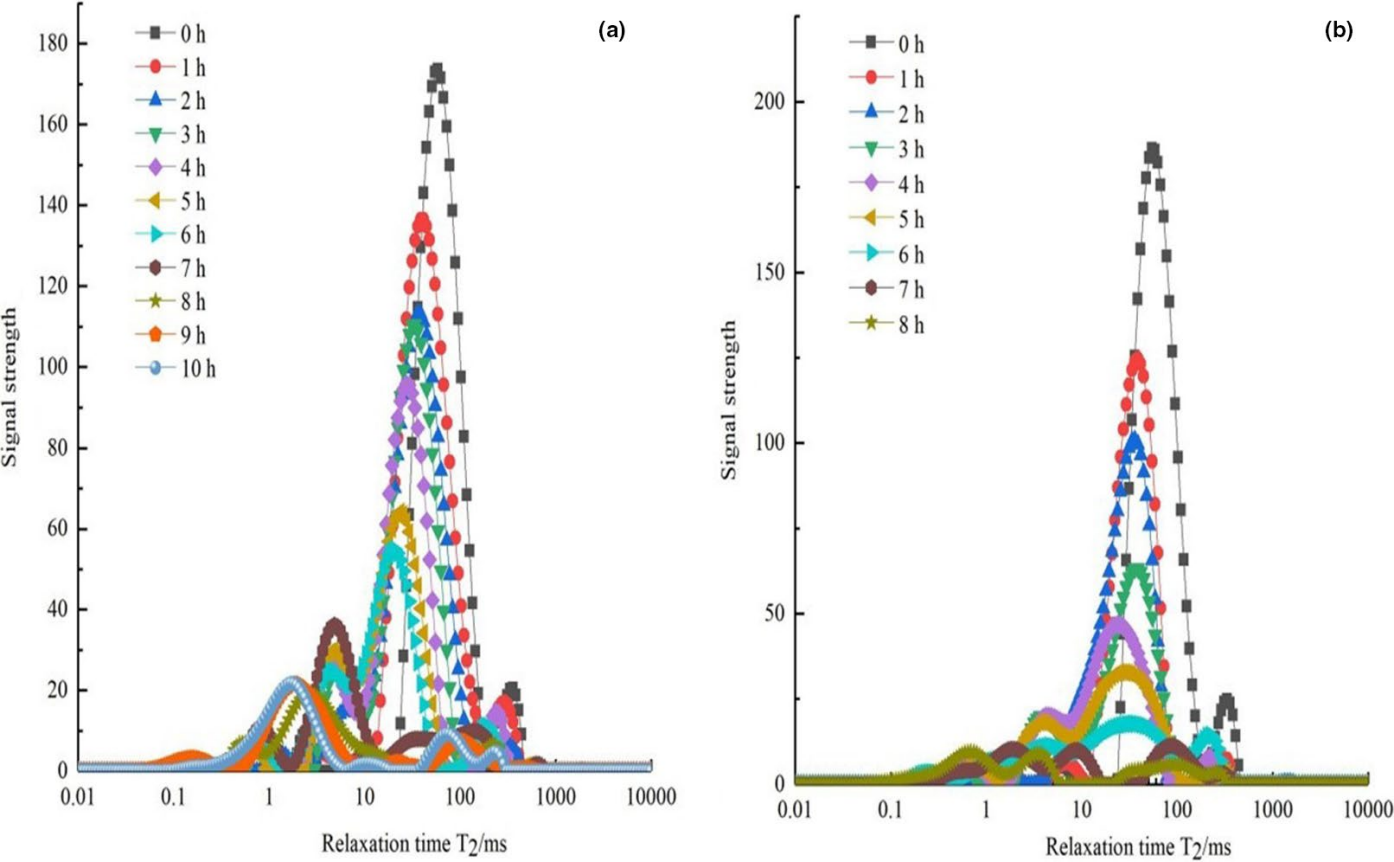
The transverse relaxation time T_2_ of tilapia fillets in different drying processes ((a) UAPOHPD processes; (b) UAPOVFHPCD processes)

Figure 4 shows the effect of different drying methods on the LF‐NMR spectra of tilapia fillets. Overall, the patterns of water migration were similar among different drying methods with the peak area initially decreasing and shifted to the left. In other words, in the early stages of drying, the entrapped water was greatly reduced, and the tilapia fillets mainly lost entrapped water. But, from Figure 4a, it can be observed that there were no obvious differences in the peaks during the process of UAPOHPD when the drying time was in the range of 8–10 hr. However, in the process of UAPOVFHPCD, the peak area of each stage showed an obviously decreasing trend (Figure 4b). In the early stages of UAPOHPD, the rate of moisture transfer from inside tilapia fillets to the surface was greater than or equal to the diffusion rate of surface moisture into the drying medium. The drying process was primarily controlled by surface vaporization (Lu et al., 2020). However, as the drying time increased, the resistance to water transfer increased. Thus, the rate that the internal moisture of the tilapia fillets migrated to the surface was less than the rate that surface moisture diffused to the drying medium. When the external drying condition remained unchanged, the surface of tilapia fillets appeared to harden, and the drying rate was further reduced (Xiao et al., 2015). Therefore, the T_2_ inversion spectra of the tilapia fillets from the late stages of UAPOHPD were not significantly different. Compared with the T_2_ inversion spectra of Figure 4a, the peaks in each stage in Figure 4b showed a significant downward trend. Since vacuum freeze‐drying was used early in the drying process, a characteristic pore structure was formed (Wang et al., 2019). When the vacuum freeze‐dried tilapia fillets were transferred to a heat pump device for drying, the pore size structure provided a means for internal moisture to escape, and the internal moisture of the tilapia fillets migrated to the surface and diffused to the drying medium through the apertures.

### 3.3 The effect of different drying methods on the moisture state of tilapia fillets

#### 3.3.1 Rupture of the cell membrane during different drying methods

Water in fresh tilapia fillets primarily distributed intercellular and intracellular, but the proportion of entrapped water is the largest. The migration of water is key for determining product quality, and intracellular water primarily migrates in two ways (Li et al., 2020). First, intracellular water can migrate into extracellular regions through intracellular pathways when materials are dried at low temperatures and the cell membranes of raw materials remain intact. Second, intracellular water can migrate through extracellular pathways when materials are dried at high temperatures and the cell membranes of raw materials rupture, thereby increasing free water content (Khan & Karim, 2017; Prothon et al., 2003).

**FIGURE 5:**
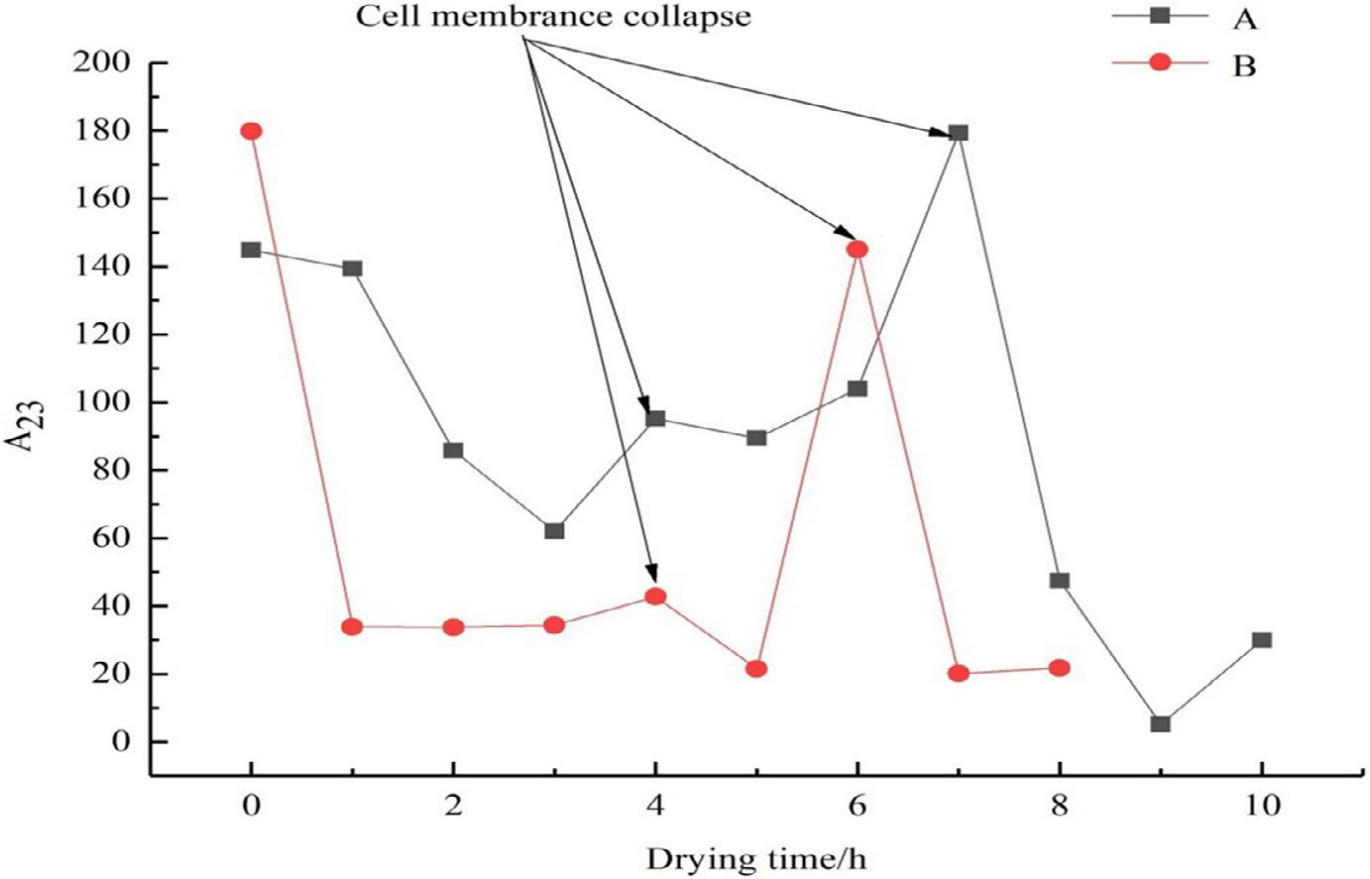
The change in the content of free water in different drying with drying time ((a) UAPOHPD; (b) UAPOVFHPCD)

**FIGURE 6:**
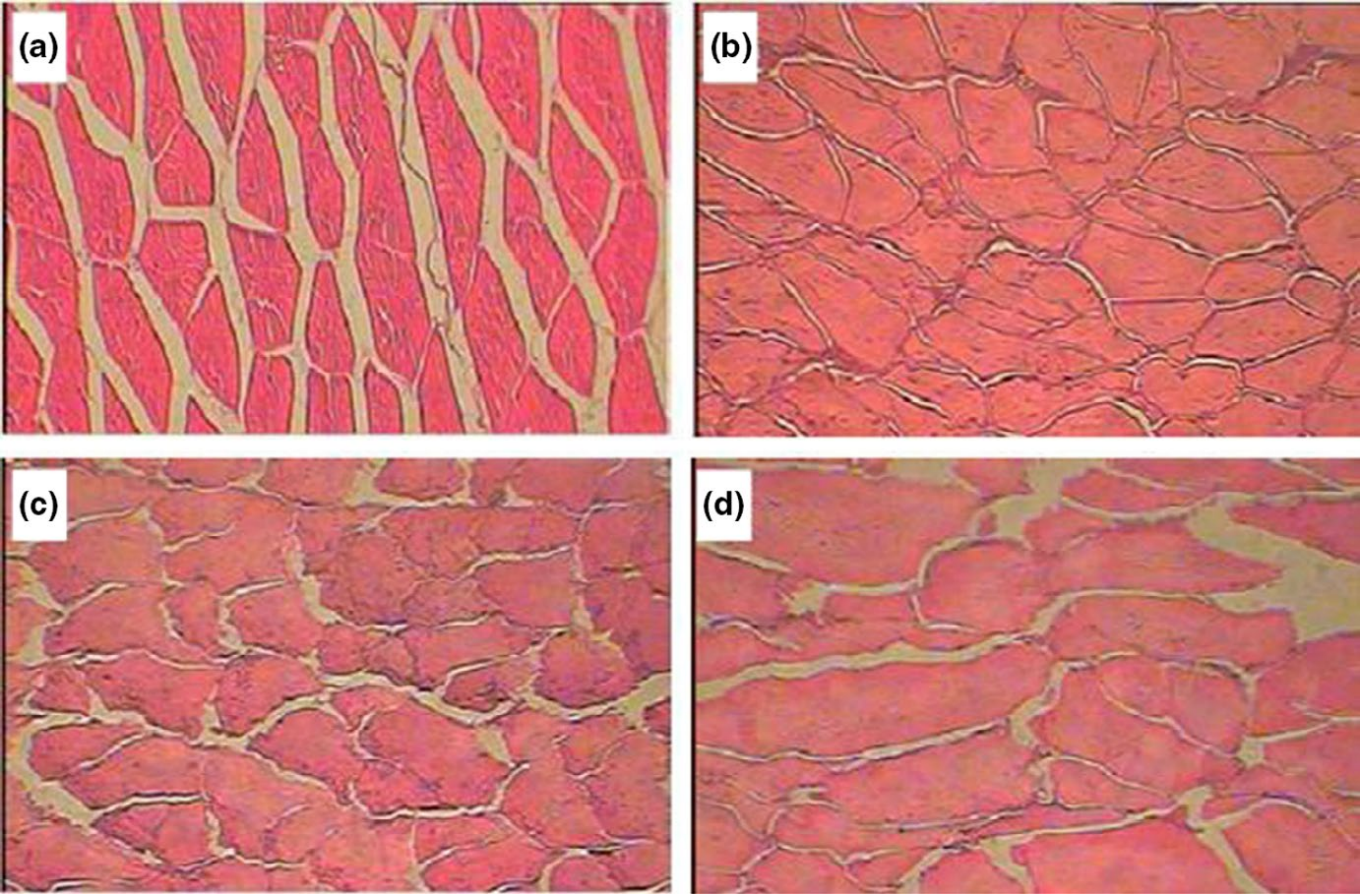
The tissue structure of tilapia fillets at different temperatures ((a) fresh fish fillets; (b) 30℃; (c) 45℃; (d) 60℃)

**FIGURE 7:**
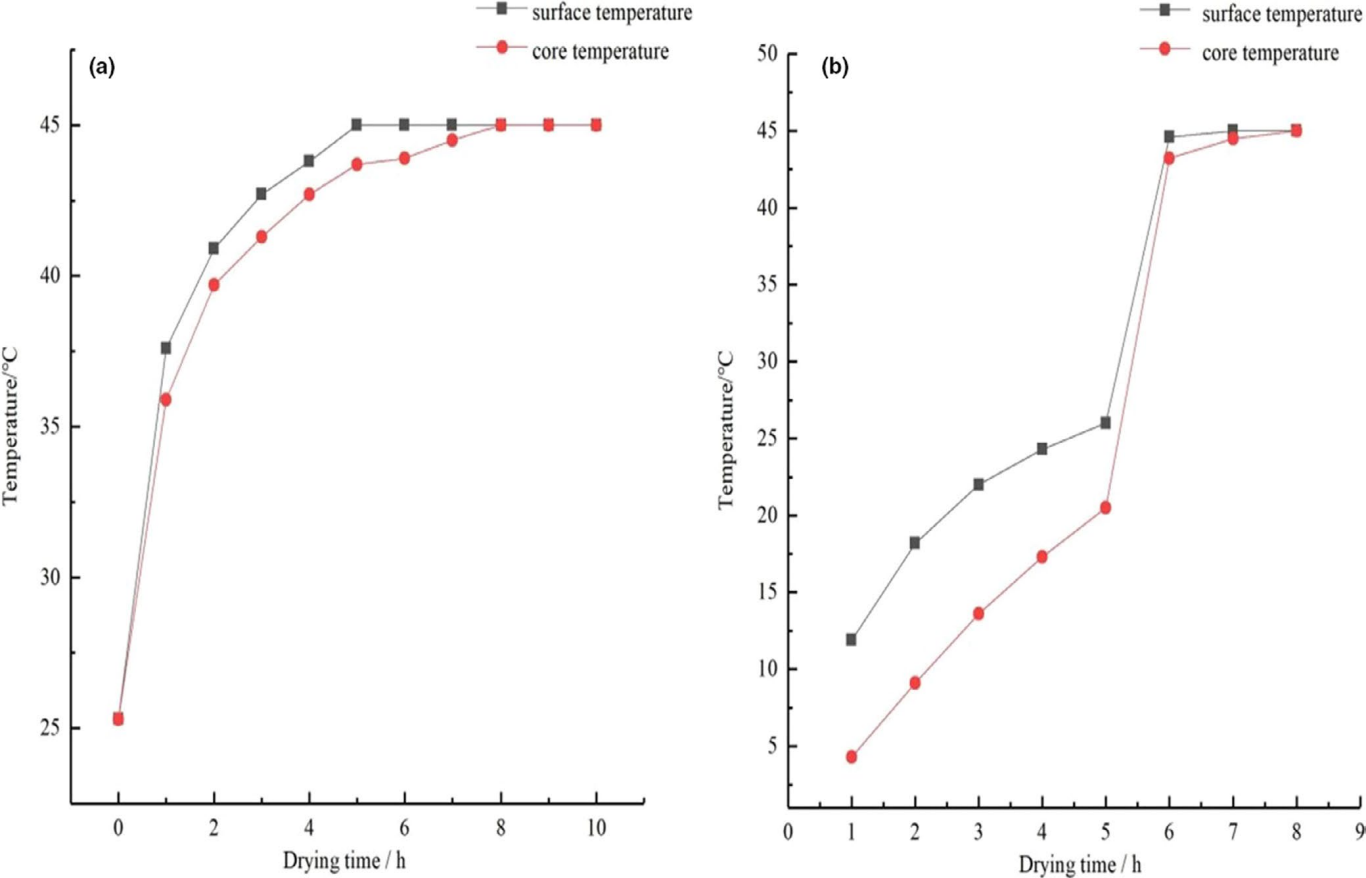
Surface and center temperatures of tilapia fillets under different drying methods ((a) UAPOHPD; (b) UAPOVFHPCD)

Previous work has shown that when plant‐based food materials are dried at temperatures below 50℃, cell membranes do not rupture; as a consequence, intracellular water flows out of the cell through pores. For example, Halder et al. (2011) dried potato chips at 45℃ and 55℃ and found that the cell membranes of potato chips maintained their integrity throughout the drying process at 45℃. In contrast, under 55℃, especially when the sample temperature was over 50℃, cell membranes broke, and intracellular water migrated out of the cell because of the rupturing of cell membranes. In addition, Khan & Karim, 2017, Khan et al. (2018) used LF‐NMR technology to study the rupturing of cell membranes during the drying process of potatoes and apples. They found that when the sample temperature was below 50℃, the cell membrane did not rupture, but when the sample temperature increased to approximately 53℃, the cell membranes on the sample surface broke. Compared with plant‐based food materials, since aquatic products are rich in protein, therefore, they are sensitive to heat. Researchers in the laboratory had discovered that the tissues of tilapia fillets were ruptured and whether fillets were pretreated by any methods, when fillets were dried at different temperatures. Figure 5 shows the change in the content of free water over time during the different drying processes. And Figure 5 is created using the data from this study, where it can be seen that there are many peaks. Figure 6 is acquired from our own experimental data. It can be seen that the cell membranes had rupturing in different degrees when tilapia fillets were dried at 30℃, 45℃, and 60℃ as shown in Figure 6 (Zheng, 2013). When the cell membranes started to rupture, as a result, the free water is the highest because the exposed intracellular water is able to readjust rapidly to become free water. Through the phenomenon, the low‐field nuclear magnetic resonance technique is used to measure the change in free water during the drying process to determine the cell membrane rupture point. Thus, in Figure 5, these peaks are represented as cell membrane rupturing point. The content of free water in tilapia fillets changed as the drying time continues to increase under different drying methods (Figure 5). From Figure 5a, it can be seen that when the drying time was in the range of 3 to 9 hr, free water appeared to initially increase and then decrease in UAPOHPD process. In order to interpret the phenomenon, the average surface and center temperature curves are presented in Figure 7a. It can be observed that at 3 hr of drying the temperature at the surface rose to 42.7℃ and the temperature at the center rose to 41.3℃ from Figure 7a; at this time, the free water in intercellular environment is migrated from the surface of the tilapia fillets and then to the environment through evaporation (Vasić et al., 2014); therefore, the curve of free water dropped. With increasing drying time, it can be observed that there is one peak at 4 hr of drying, and the peak is represented as cell membrane collapse point (Khan & Karim, 2017). The reason might be due to the cell membranes close to the surface of the samples will start to rupture first; when the temperature at the surface rose to 43.8 ℃ and the temperature at the center rose to 42.7℃, the temperature had been used for tropical species (Mujaffar et al., 2005); however, the aquatic products are sensitive to heat, which should be dried at 27℃ (Ying et al., 2011). As a result, the entrapped water (intracellular water) is able to readjust rapidly to become free water, which made the curve of free water increasing (Figure 5a). As the drying time increased, the free water is gradually decreasing due to its migration up to the surface and then to the environment through evaporation, but it can be observed that there is a peak that emerged when the drying time was 7 hr. With increasing drying time, the continuous penetration of heat energy from surface to the entire sample temperature from surface to the temperature inside the sample is reached about 45°C. (Kumar et al., 2012). It can be observed that the temperature at the surface rose to 45℃ and the temperature at the center rose to 44.5℃ at 7 hr of drying from Figure 7a, and the entire sample temperature from surface to the temperature inside the sample reached about 45°C. There is a peak at this temperature, and it might be due to the entire muscle fiber network of the tilapia fillets tend to shrink and break, which increases the content of the free water (Ling et al., 2020); therefore, it can be assumed that the peak of the free water curves is represented as cell membrane collapse point. However, when the drying time was greater than 7 hr, the center temperature and surface temperature of the samples were the same, and the remaining free water in the samples continuously diffused into the drying medium, resulting in a decreasing trend. At the end of drying, the peak area of free water showed an increasing trend because of the oil produced from tilapia fillets.

From Figure 5b, there was no significant difference in the free water content during the early stages of UAPOVFHPCD, because the tilapia fillets were sublimated directly under low temperature and low pressure, when the drying time was in the range of 1 to 3 hr. But there was a peak at 4 hr of drying (Figure 5b). Since when tilapia fillets were dried in a state of low pressure for a long time, there was the expansion force of the cells in tilapia fillets, which made a small number of cells rupture and increasing content of the free water (Liu et al., 2012). However, compared with UAPOHPD, the peak of UAPOVFHPCD was lower. Because the entire temperature of the tilapia fillets was lower at 4 hr of drying, only a few cells were ruptured. After vacuum freeze‐drying, tilapia fillets became porous structures, because a vacuum environment promoted the formation of porous structures inside the tilapia fillets (Wang et al., 2019). When the tilapia fillets were transferred to the heat pump device for drying, the temperature was quickly diffused to the center of the tilapia fillets through the apertures. The overall temperature of the tilapia fillets increased over a short period (Figure 7b). It can be observed that the temperature at the surface rose to 44.6℃ and the temperature at the center rose to 43.5℃ at 6 hr of drying from Figure 7b. In addition, it can be observed that there was one peak at 6 hr of drying and the free water is the highest (Figure 5b). The reason could be due to the cell membranes close to the surface of the samples rupturing; when the temperature at the surface was 44℃ and the temperature at the center was 43.5℃ (Figure 7b), the exposed entrapped water (intracellular water) is able to readjust rapidly to become part of the free water; thus, the curve of free water increases. With increasing drying time, free water is gradually decreasing due to the free water evaporated during the drying process. However, in the late drying period, there was no significant difference in the free water content, because the entrapped water had all been converted to free water to evaporate.

#### 3.3.2 The effect of different drying methods on the migration of entrapped water in tilapia fillets

**FIGURE 8:**
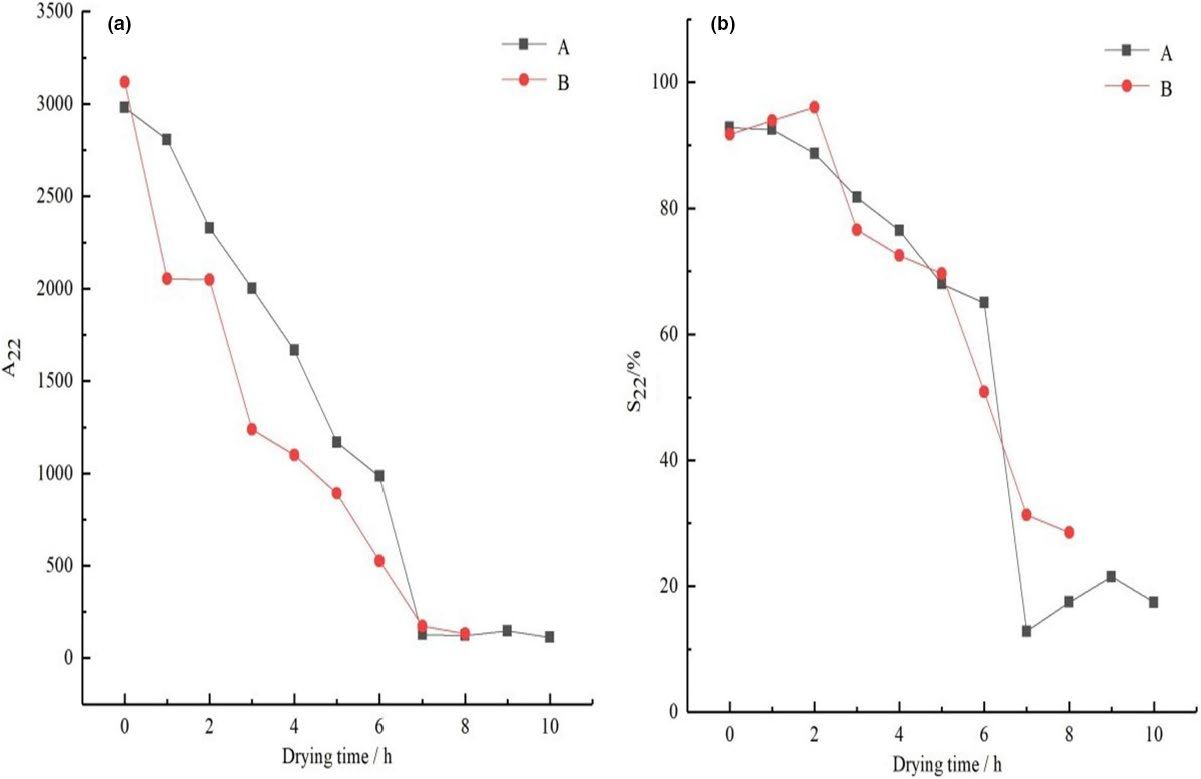
The effect of different drying methods on the migration of entrapped water in tilapia fillets ((a) the peak area of the entrapped water; b: the ratio of the entrapped water to the total peak area; (a) UAPOHPD; (b) UAPOVFHPCD)

Figure 8 shows that the entrapped water of tilapia fillets was changed as drying time under different drying methods. The A_22_ of tilapia fillets subjected to UAPOVFHPCD was smaller than the A_22_ of tilapia fillets subjected to UAPOHPD, when the drying time was equal (Figure 8a). However, the reasons for the decline in A_22_ were different. In the process of UAPOHPD, the A_22_ of the samples declined because the tilapia fillets were in contact with oxygen for long periods during the drying process; thus, myosin and sarcoplasmic were oxidated, which resulted in decrease in water‐holding capacity (Guan et al., 2012; Xie et al., 2014). Promoting the diffusion of entrapped water to the surface was key, as this caused A_22_ and S_22_ to show decreasing trends. However, when the drying time was greater than 7 hr, A_22_ showed a subtle trend: After drying for 7 hr, almost all of the intracellular water had exited the cells and became free water. Therefore, there was no significant change in A_22_ as the tilapia fillets continued to be dried.

Compared with UAPOHPD, the A_22_ of the samples showed a consistent downward trend during the process of UAPOVFHPCD. In the vacuum freeze‐drying stage, the water inside the sample froze rapidly, and the water formed fine ice crystals that were sublimated. At the same time, pores were formed inside the samples as the ice crystals sublimated (Wang et al., 2019). In the later stages of heat pump drying, the water of the samples diffused through the pores, resulting in a decreasing trend in A_22_ and S_22_ during the drying process.

#### 3.3.3 The effect of different drying methods on the migration of bound water in tilapia fillets

**FIGURE 9:**
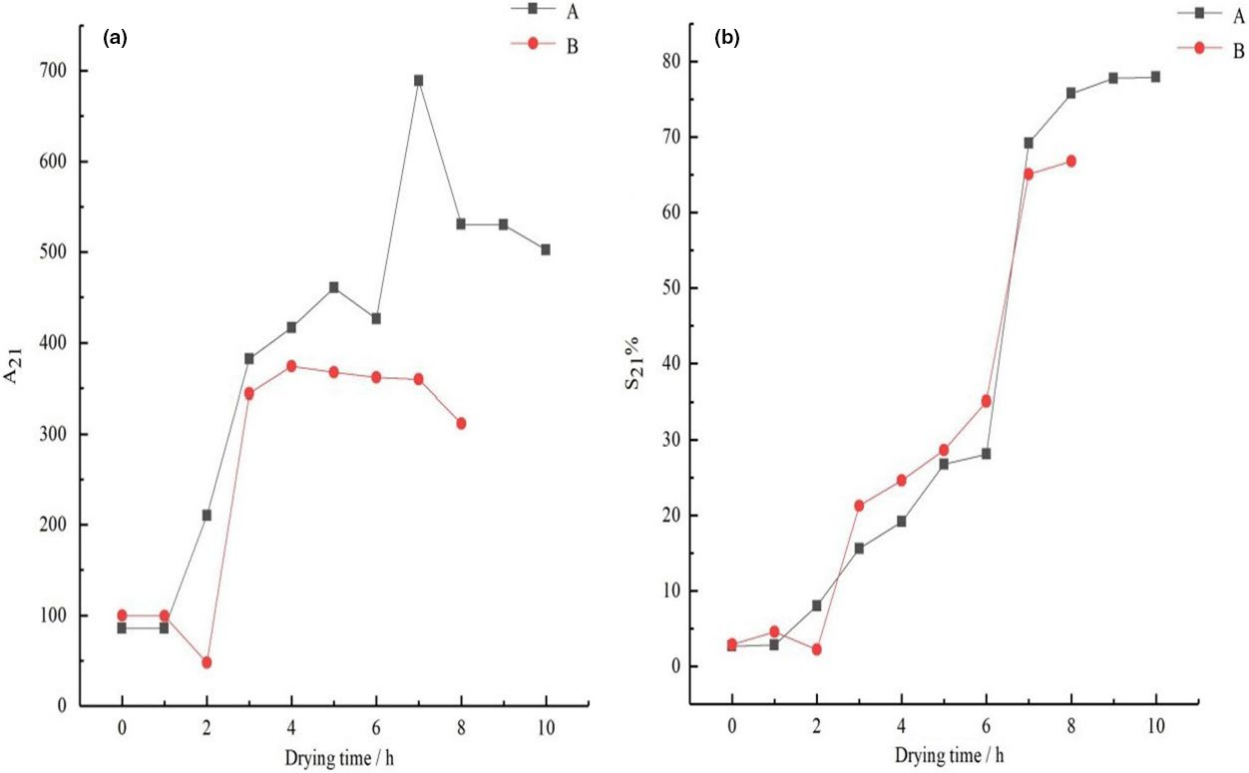
The effect of different drying methods on the migration of bound water in tilapia fillets ((a) the peak area of the bound water; (b) the ratio of the bound water to the total peak area; (a) UAPOHPD; (b) UAPOVFHPCD)

Bound water refers to the water that is most closely bound to proteins (Pearce et al., 2011). Changes in bound water are primarily affected by water–protein interactions (Sette et al., 2016), as water can exchange protons with proteins and the denaturation and aggregation of proteins can affect the proton exchange between water and proteins (Einarsdottir et al., 2014; Rao et al., 2016). The peak area of the bound water of tilapia fillets initially increased and then decreased under different drying methods (Figure 9). From Figure 9a‐A, it can be seen that during the process of UAPOHPD, the A_21_ of samples showed a decreasing trend when they were dried for 5 hr. However, when the drying time was extended to 6 hr, A_21_ increased significantly. Continued drying resulted in decreases in A_21_, as the entrapped water from the early stages of drying continued to decrease, leading to a decline in cell activity, an increase in the concentration of cells in tilapia fillets, and the binding of some water molecules through hydrogen bonds to the protons of proteins (Xu and Yu, 2014). Because the entrapped water can pass through the cell membrane, some water molecules bind to the hydrophilic groups of proteins (Einhorn‐stoll & Hatakeyama, 2012). During the drying process, some proteases and organics of tilapia fillets were decomposed, which converted the bound water to entrapped water, thereby reducing the bound water content. However, when the drying time of UAPOVFHPCD was within the range of 4–7 hr, A_21_ tended to be flat (from Figure 9a‐B). Because the materials were dried by vacuum freeze‐drying during the early stages of drying, the entrapped water of the materials was frozen, forming ice crystals that were directly sublimed. When the tilapia fillets were transferred to the heat pump for drying, the entrapped water content of the tilapia fillets was reduced; as a result, there was virtually no conversion of entrapped water to bound water. S_21_ showed an increasing trend as the drying time increased (Figure 9b). Thus, the moisture during this stage was relatively stable and was difficult to remove during the drying process. Therefore, the drying effect of tilapia fillets was virtually unaffected during the drying process.

### 3.4 Moisture distribution

**FIGURE 10:**
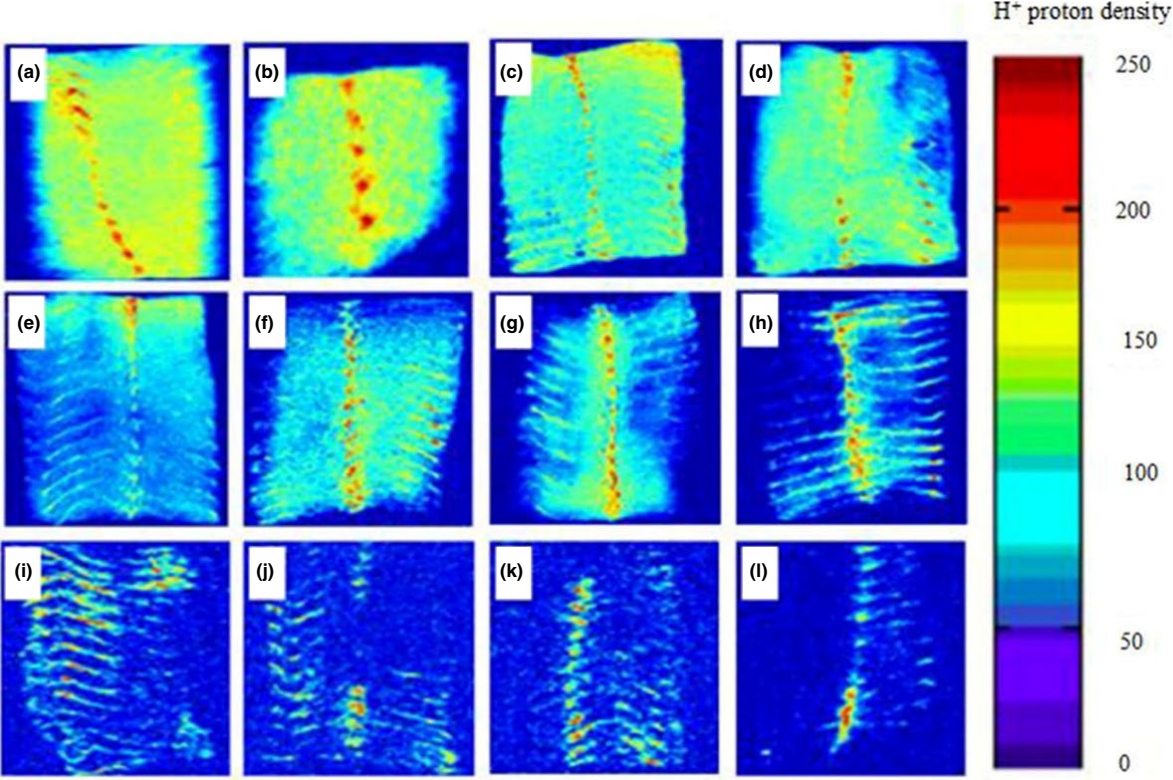
Hydrogen proton images of tilapia fillets during the drying process of UAPOHPD ((a) fresh samples; (b) 0 hr; (c) 1 hr; (d) 2 hr; (e) 3 hr; (f) 4 hr; (g) 5 hr; (h) 6 hr; (i) 7 hr; (j) 8 hr; (k) 9 hr; (l) 10 hr)

**FIGURE 11:**
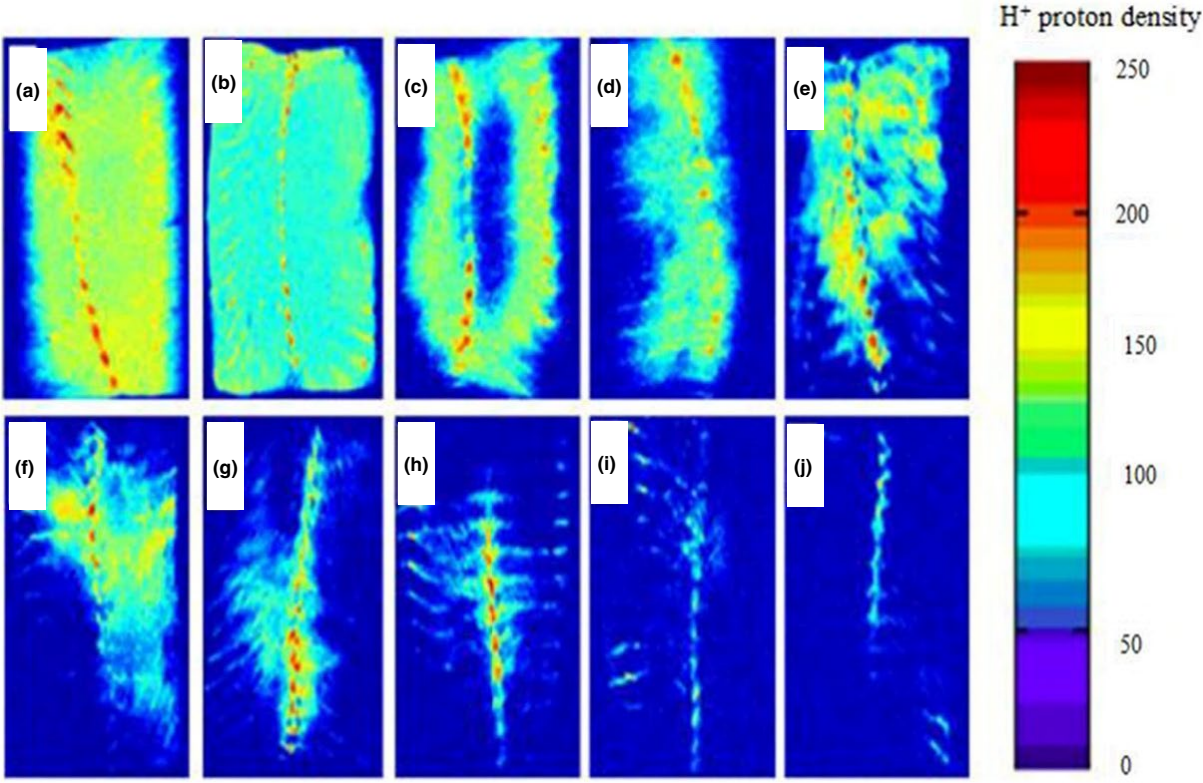
Hydrogen proton images of tilapia fillets during UAPOVFHPCD ((a) fresh samples; (b) 0 hr; (c) 1 hr; (d) 2 hr; (e) 3 hr; (f) 4 hr; (g) 5 hr; (h) 6 hr; (i) 7 hr; (j) 8 hr)

MRI can form a two‐dimensional proton density map that can directly detect the content and distribution of water or oil in samples. The signal intensity of the sample is directly proportional to its internal moisture content. The brighter blue the image, the higher the moisture content of the tilapia fillet. In contrast, when the moisture content is lower, the picture is close to the background color (blue), as shown in the legend on the right side of Figure 10a–l and Figure 11a–j. When the red value is larger, the density of H^+^ protons and the water content are higher. Thus, changes from red to blue indicate gradual decreases in the water content of samples. Tilapia fillets not only contain high quantities of water and protein but also contain fat. The protons provided by fat will also make the MRI images brighter. The distribution of water in the fresh tilapia fillets was uniform. In addition, under different drying conditions, the water content of the tilapia fillets moved from outside to inside as the length of the drying time increased, and the MRI images became increasingly blurred.

Compared with the MRI diagrams in Figure 10a–l, the MRI diagrams of Figure 11a–j were darker over the same drying period, indicating that the moisture content of the samples was reduced. This difference stems from the fact that UAPOVFHPD was used to dry tilapia fillets. This technique facilitated the formation of pores during the early stages of drying, which favored the diffusion of moisture and increased the rate of drying relative to the rate documented for UAPOHPD. In addition, the tilapia fillets were dried in different ways until drying was complete, as a red region remained in the MRI images. This lingering red region stemmed from the production of oil by tilapia fillets during the drying process. However, in the process of UAPOHPD, the average temperature during the drying process was higher than that for UAPOVFHPCD, leading to the generation of more fat during the drying process. Therefore, when the drying was complete, the red part of the MRI images for the UAPOHPD was brighter than that for the UAPOVFHPCD.

## 4 CONCLUSION

1. The peak area decay rate of free water, entrapped water, and bound water in the UAPOVFHPCD process was significantly more rapid than that of UAPOHPD, which indicated that the speed of UAPOVFHPCD was faster than the speed of UAPOHPD.
2. Imaging technology revealed that the internal moisture of the sample was constantly removed and that the exudation of fat constantly increased as the drying time increased. Reducing the average drying temperature can reduce the effect of fat exudation on the drying process.
3. This paper presents the migration of water in different environments. The proportion of intercellular and intracellular water in tilapia fillets at different stages of drying was measured by low‐field nuclear magnetic resonance technique. The study found that the cell membranes rupture at different stages of drying rather than collapsing all at once. The rupture of cell membranes mainly depends on the penetration of heat energy. The study also found that when cell membrane ruptures, the exposed intracellular water is able to readjust rapidly to become free water; at this time, the free water is the highest. Understanding the law of cell membrane rupturing during the drying process，which will improve the quality of the dried products. In addition, the drying rate will be accelerated when understanding the law of cell membrane rupturing during the drying process and improving the drying conditions, which can provide a theoretical basis for solving the low temperature drying rate problem of tilapia fillets and similar aquatic products.

## CONFLICT OF INTEREST

The authors declare no conflict of interest.

## ETHICAL APPROVAL

This investigation did not involve human or animal testing.

## Data Availability

The research data are not shared.
